# 

**Published:** 1886-10

**Authors:** 


### CONTENTS—OCTOBER, 1886.

ORIGINAL ARTICLES—An Obscure Case of Peri-Uterine Cellulitis, By Dr. W. C. Fisher, of Galveston ..... 133
Experience of a Medical Expert. By Dr. F. A. Schmidt, Schulenburg ..... 138
Craniotomy, a Case of. By Dr. Peyton Turner, of Abilene 141
A case of Hour-Glass Contraction After the Third Stage of Labor. By R. W. Skipper, M. D., Bend, Texas ..... 144
CORRESPONDENCE—Letter from Prof. Keyser, Dean of the Medico-Chirurgical College, Philadelphia ..... 145
Darwinian Theory vs. Special Creation. Letter from Dr. Park, of Waco ..... 146
A Correction. Note from Dr. Hadra ..... 149
A "New Way to Take the Temperature." Note from Dr. Sessions ..... 150
CULLINGS FROM CONTEMPORARIES—Dr. Delafield's Address: Extract ..... 151
Small Doses Which are Effectual ..... 152
Lanolin and Ichthyol ..... 153
SOCIETY NOTES—Physicians' Mutual Benefit Association of Texas 154
Report of the Organization of the Central Texas Medical Association ..... 156
EDITORIAL—Jury Trial of the Pauper Insane ..... 159
A Prophecy ..... 164
Tait's Operation in Texas ..... 166
Alas! Poor Yorick ..... 168
NECROLOGICAL—In Memoriam—Hays White Moore, M. D. ... 170
Died—Albert Welch, M. D ..... 173
EDITORIAL NEWS AND MISCELLANY ..... 174
ADVERTISERS' NOTICES ..... 176

